# Quantitative Determination of Fatty Acids in Marine Fish and Shellfish from Warm Water of Straits of Malacca for Nutraceutical Purposes

**DOI:** 10.1155/2013/284329

**Published:** 2012-12-23

**Authors:** Nurnadia Abd Aziz, Azrina Azlan, Amin Ismail, Suryati Mohd Alinafiah, Muhammad Rizal Razman

**Affiliations:** ^1^Department of Nutrition and Dietetics, Faculty of Medicine and Health Sciences, Universiti Putra Malaysia, 43400 Serdang, Selangor, Malaysia; ^2^Laboratory of Halal Science Research, Halal Products Research Institute, Universiti Putra Malaysia, 43400 Serdang, Selangor, Malaysia; ^3^Institute for Environmental and Development (LESTARI), Universiti Kebangsaan Malaysia, 43600 UKM Bangi, Selangor, Malaysia

## Abstract

This study was conducted to quantitatively determine the fatty acid contents of 20 species of marine fish and four species of shellfish from Straits of Malacca. Most samples contained fairly high amounts of polyunsaturated fatty acids (PUFAs), especially alpha-linolenic acid (ALA, C18:3 n3), eicosapentaenoic acid (EPA, C20:5 n3), and docosahexaenoic acid (DHA, C22:6 n3). Longtail shad, yellowstripe scad, and moonfish contained significantly higher (*P* < 0.05) amounts of eicosapentaenoic acid (EPA), docosahexaenoic acid (DHA), and alpha-linolenic acid (ALA), respectively. Meanwhile, fringescale sardinella, malabar red snapper, black pomfret, Japanese threadfin bream, giant seaperch, and sixbar grouper showed considerably high content (537.2–944.1 mg/100g wet sample) of desirable omega-3 fatty acids. The polyunsaturated-fatty-acids/saturated-fatty-acids (P/S) ratios for most samples were higher than that of Menhaden oil (P/S = 0.58), a recommended PUFA supplement which may help to lower blood pressure. Yellowstripe scad (highest DHA, *ω* − 3/*ω* − 6 = 6.4, P/S = 1.7), moonfish (highest ALA, *ω* − 3/*ω* − 6 = 1.9, P/S = 1.0), and longtail shad (highest EPA, *ω* − 3/*ω* − 6 = 0.8, P/S = 0.4) were the samples with an outstandingly desirable overall composition of fatty acids. Overall, the marine fish and shellfish from the area contained good composition of fatty acids which offer health benefits and may be used for nutraceutical purposes in the future.

## 1. Introduction

Fish and shellfish are widely accepted as highly nutritious and healthy foods. However, people usually think that different types of fish are of similar nutritional value, and fish selections are made only based on availability, freshness, flavor, and other physical factors [1]. Based on the Malaysian Adult Nutrition Survey (2002-2003), the prevalence of daily consumption of marine fish among rural and urban adults were quite high at 51% and 34%, respectively [2]. Therefore, it is crucial to increase the awareness of different nutrient contents of fish and shellfish species by providing complete nutritional value information, especially for fatty acid content, which is associated with various health-related effects.

Nutritional analysis of fatty acids can be classified as qualitative and quantitative. Qualitative analysis of fatty acid produces data regarding the fatty acid composition in the form of percentages of total fatty acids (% of total fatty acids). Meanwhile, quantitative analysis is able to quantify the actual amount (weight) of each fatty acid that is present in the food. Quantitative data are often presented in the form of weight of the fatty acid per weight of food or fat (e.g., mg/g oil). Currently, there are limited qualitative fatty acid data on marine fish and shellfish from warm water area; meanwhile, no quantitative data are available especially as a mean to utilize source of natural product for nutraceutical purposes.

Reliable analytical data are prerequisite for correct interpretation of findings in nutritional content analysis, since unreliable results may lead to over- or underestimations, false interpretations, and unwarranted conclusions [3]. Thus, validation procedures of analytical methods were also performed to provide reliable quantitative data on the fatty acid contents of various species of local marine fish and shellfish.

## 2. Materials and Methods

### 2.1. Chemicals and Reagents

All chemicals and reagents used for analysis were of analytical and gas chromatography (GC) grade. Menhaden oil, 37 components FAME mix 47885-U (Supelco, Germany), and tridecanoic acid (C13, internal standard, Sigma, USA) were used as standards in the fatty acid content analysis.

### 2.2. Preparation of Sample

**Figure 1:**
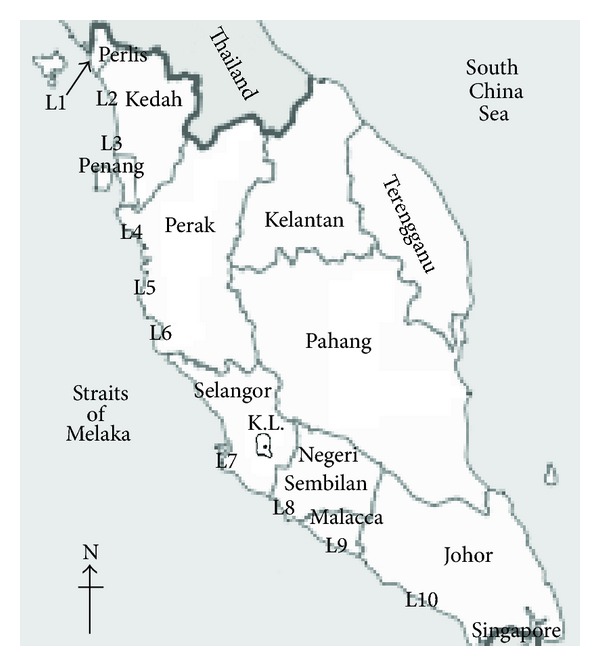
Location of sample collection sites.

A stratified random sampling procedure was used as it was the most suitable method in database work [4]. To ensure representativeness, ten fish landing areas along the Straits of Malacca were identified with the help of Malaysian Fisheries Development Authority (LKIM). The locations are marked as L1 through L10, respectively (Figure 1).

At each of the collection sites, available samples were collected randomly according to species. All samples were from free-roaming fish and shellfish; and they were collected fresh (caught within a period of 0 to 36 hours). All samples were immediately placed on ice, kept cold, and transported in polystyrene boxes to maintain freshness. Upon arrival at Universiti Putra Malaysia, the temperature of ice boxes was checked to ensure samples were still within the range of −4°C to 0°C. Then, samples for nutrient determination were individually measured for total body weight and length. Only samples of a weight within the narrow range for each species were included as primary samples (Table 1). Then, the samples were beheaded, gutted, washed, and filleted. These primary samples were placed in sealed plastic bags and frozen at −20°C. The actual degree of freshness of samples during transportation and time of analysis may not be assured as there was no analysis of freshness or quality index of samples was done. However, appropriate precautions were performed to sustain freshness of samples and minimize oxidation throughout the study by performing procedures in chillers' room (4°C) and under minimal light exposures.

A small-scale experiment performed independently showed insignificant differences in nutrient contents of samples from different locations. This observation justified that units of primary samples can be combined or composited by geographical locations to minimize the number of analytical measurements and yet represent the contribution of the unit to the estimate of central tendency [4]. Thus, before analysis, three composite samples were prepared by mixing individual samples (*n* = 8–12, whole fillets) of the same weight for each species. Individual samples from L1, L2, L3, and L4 constituted as Composite 1; individual samples from L5, L6, and L7 constituted as Composite 2; while individual samples from L8, L9, and L10 constituted as Composite 3 (Figure 1). All composite samples were analyzed separately and data presented are the mean values of each of the species.

### 2.3. Extraction of Fat

Extraction of fat was done following Bligh and Dyer [6], with slight modifications by Kinsella et al. [7]. Representative samples of fish filets (30 g) were homogenized in a Waring blender for 2 min with a mixture of methanol (60 mL) and chloroform (30 mL). One volume of chloroform (30 mL) was added to the mixture and after blending for additional 30 seconds, distilled water (30 mL) was added. The homogenate was stirred with a glass rod and filtered through Whatman No. 1 filter paper on a Buchner funnel with slight suction. The filtrate was transferred to a separatory funnel. The lower clear phase was drained into a 250 mL round-bottom flask and concentrated with a rotary evaporator at 40°C. To minimize oxidation, the extracted lipids were kept in solvents containing 0.05% butylated hydroxytoluene (B.H.T.), in glass bottles, flushed with nitrogen, and wrapped with aluminium foils to avoid any light exposure. Then, the bottles were stored immediately at −20°C and only being taken out from freezer just before analysis.

### 2.4. Analysis of Fatty Acid Content

Lipid samples were converted to their constituent FAMEs following the method used in previous study [8]. Approximately 25 mg (±0.1 mg) of oil was weighed and added with 1.5 mL of NaOH 0.50 M in methanol in a 15 mL capped centrifuge tube. The mixture was heated in a water bath at 100°C for 5 min and then cooled at room temperature. The mixture was added with 2.0 mL of boron trifluoride (BF_3_, 12%) in methanol and heated again in a water bath at 100°C for 30 minutes. Next, the tube was cooled in running water at room temperature before adding 1 mL of isooctane. It was vigorously stirred for 30 seconds before adding 5.0 mL of a saturated sodium chloride solution to facilitate the phase separation. The esterified sample was placed in a refrigerator and left to rest for better phase separation. After collecting the supernatant, another 1.0 mL of isooctane (containing 0.05% B.H.T. as antioxidant) was added into the tube and stirred. The supernatant was collected and added to the previous fraction. The sample was concentrated to a final volume of 1.0 mL for later injection into the gas chromatograph. As precautions, amber vials were used in order to minimize oxidation during analysis.

*Analysis of Gas Chromatography (GC)*. Analysis of methyl esters was performed by a capillary gas chromatograph model Agilent 6890 (USA Agilent Technology) equipped with a split-splitless injector, flame ionization detection (FID) system used to separate and quantify each FAME component. FAMEs were separated using a highly polar HP88 column (Agilent, USA) column (100 m length × 0.25 mm I.D. × 0.2 *μ*m D.F.). Carrier gas was helium at a linear velocity of 30.0 mL/min. Split injection with a split ratio (volume of gas passing to waste: volume of gas passing down the capillary column) of 10 : 1 and 99.9 mL/min split flow was used. The operating conditions were 250°C injection port, 250°C flame ionization detector, and 200°C column temperature. Compounds were identified by comparison of the retention times of 37 components FAME mix 47885-U (Supelco, Germany) and Menhaden oil standards (Supelco, Germany).

*Quantification of Fatty Acids*. The concentration of fatty acids in mg/g of total lipids was measured against tridecanoic acid methyl ester (C13:0, Sigma, USA) as an internal standard. This followed procedures described in a previous study [6] with slight modifications; in which the Empirical Response Factor (*R*_*i*_) of FID (flame ionization detector) was calculated and used instead of theoretical response factor (*C*_FX_). Calculation of the empirical response factor (*R*_*i*_) was done as described in the literature [9]. The following formulae were used in the quantification of fatty acids: 

(i) (1) $$\begin{array}{c} \text{empirical}\;\text{response}\;\text{factor}\;\;\left(R_{i}\right)=\frac{\left(\text{Psi}\right)\left(\text{WISm}\right)}{\left(\text{PsIS}\right)\left(\text{Wi}\right)}, \end{array}$$

where Psi: peak area of individual fatty acids in mixed FAME standard solution, PsIS: peak area of fatty acid internal standard in mixed FAME standard solution, WISm: weight of fatty acid internal standard in mixed FAME standard solution, and Wi: weight of individual FAME in mixed FAME standard solution;

(ii)  
  (2) $$\begin{array}{c} \text{fatty}\;\text{acid}\;\left(\text{mg}/\text{g}\right)\;\text{total}\;\text{lipid}=\frac{\left(\text{AX}\right)\left(\text{WIS}\right)\left(R_{i}\right)\left(1000\right)}{\left(\text{AIS}\right)\left(\text{WX}\right)\left(1.04\right)}, \end{array}$$

where AX: peak area of fatty acid, AIS: peak area of internal standard (IS) WIS: weight (mg) of IS added to the sample (mg), WX: sample weight (mg), Ri: empirical response factor, and 1.04: conversion factor necessary to express results in mg fatty acid/g oil (rather than as methyl ester).

### 2.5. Analysis of Method Validation

*Linearity Test*. Three fatty acid standards, myristic acid, heptadecanoic acid, and linoleic acid were prepared at different concentrations: myristic acid (0.4, 2.0, and 4.0 mg/mL), heptadecanoicacid (0.4, 2.0, and 4.0 mg/mL), linoleic acid (0.5, 1.0, and 5.0 mg/mL). These fatty acid standards were used as they represent both saturated and unsaturated fatty acids present in samples. Calibration curves were formed for each of the compounds. The linearity parameters, which included linear regression (*y* = *mx* + *c*) and the correlation coefficient (*R* ^2^) were obtained from the linear relationship between the peak area and the concentrations of the fatty acid standards.

*Precision Test*. Two precision tests of repeatability (within-day) and reproducibility (between-day) were performed. Two fatty acid standards were quantified in three randomly selected samples in both tests. For repeatability (within-day precision), four replicates of each sample were analyzed in a single day using the same procedures as in the fatty acid analysis. Data are reported as relative standard deviations (RSD) of four replicates, with minimum and maximum values of both palmitic and linolenic acids quantified in each sample. For reproducibility (between-day precision), samples were analyzed using the same procedures as in the fatty acid analysis on three different days, representing three replicates of each sample. Palmitic acid and linolenic acids were quantified and presented as relative standard deviations (RSD) of three replicates, with minimum and maximum values of each fatty acid.

*Recovery Test*. The recovery of the method was determined using three different concentrations (0.4, 2.0, 4.0 mg/mL) of myristic acid, heptadecanoic acid, and stearic acid standards. Two samples were randomly selected and used for the recovery analysis of each of the fatty acid standards. During the sample preparation, each standard was added together with lipid extract and internal standards and prepared following the same procedures used in normal sample preparation. Recoveries of different standards were performed separately to avoid overlapping of peaks in chromatograms which could lead to biased results. Recoveries of fatty acid standards at different concentrations were determined by comparing the content of the fatty acids in samples with and without the addition of the standard. Data are presented as the percentage of recovery.

*Statistical Analysis*. Data were analyzed using SPSS (Scientific Package of Social Science) version 17.0. The mean, standard deviation (SD), and one-way ANOVA test followed by Tukey post-hoc analysis were performed to compare differences in the mean fatty acid contents of different species of fish and shellfish. Bivariate correlation (Pearson's *r*) was performed to explore the relationships between different fatty acid classes in samples.

## 3. Results and Discussion

### 3.1. Fatty Acid Content in Samples

Data from this study are reported in the form of milligrams per 100 grams of wet muscle. Tables 2, 3, and 4 show the sample content of saturated, monounsaturated, and polyunsaturated fatty acids, respectively.

Based on the previous findings, the studied fish samples can be categorized into lean-, low-, medium-, and high-fat fish based on the fat content [5]. Among the lean-fish, hardtail scad was high in saturated fatty acids (SFAs), with palmitic acid as the highest (531.7 mg/100 g wet sample) (Table 2). The monounsaturated fatty acid (MUFA) of the fish was 121.2 mg/100 g wet sample; with fairly high oleic acid (C18:1n9c) and heptadecenoic acid (C17:1), at 63.9 and 45.7 mg/100 g wet sample, respectively (Table 3). For PUFA, hardtail scad contained quite high amounts of DHA (C22:6n3), at 196.0 mg/100 g wet sample, followed by ALA (C18:3n3) at 117.3 mg/100 g wet sample (Table 4). Meanwhile, malabar red snapper was higher in PUFA, characterized by high DHA (C22:6n3) at 209.9 mg/100 g wet sample, followed by ALA (C18:3n3) at 335.0 mg/100 g wet sample (Table 4). For SFA, palmitic acid was the dominant fatty acid (373.7 mg/100 g wet sample), followed by caprylic acid at 112.8 mg/100 g wet sample (Table 2). The MUFA content was 141.8 mg/100 g wet sample, with fairly high oleic acid (C18:1 n9c) at 119.5 mg/100 g wet sample (Table 3).

Among the low-fat fish, yellowstripe scad contained a very high PUFA content, with significantly higher (*P* < 0.05) levels of DHA (782.1 mg/100 g wet sample) compared to other samples (Table 4). The DHA amount was about 2.7 times higher than Japanese threadfin bream (2nd highest DHA, 293.0 mg/100 g wet sample); and about 86.6 times higher than long-tailed butterfly ray (lowest DHA, 9.0 mg/100 g wet sample). Additionally, yellowstripe scad also had a high ALA (C18:3n3) level at 338.1 mg/100 g wet sample. For SFA and MUFA, the most dominant fatty acids were palmitic acid (560.8 mg/100 g wet sample) and oleic acid (188.8 mg/100 g wet sample), respectively (Tables 2 and 3).

The medium-fat fish, moonfish, showed palmitic acid as the most prominent fatty acid (1308.0 mg/100 g wet sample) (Table 2). This was followed by *α*-linolenic acid (C18:3n3) at 1046.8 mg/100 g wet sample, which was significantly higher (*P* < 0.05) compared to the others (Table 4). Other *ω* − 3 fatty acids, such as eicosatrienoic acid (C20:3n3), EPA (C20:5n3), and DHA (C22:6n3) were at 257.6, 176.7, and 122.6 mg/100 g wet sample, respectively. Its *ω* − 6 fatty acid content, linolelaidic acid (C18:2n6t, 637.7 mg/100 g wet sample) and linoleic acid (C18:2n6c, 215.3 mg/100 g wet sample), were the second highest after level in longtail shad (linolelaidic acid at 2254.1 mg/100 g and linoleic acid at 1327.4 mg/100 g wet sample), respectively. Meanwhile, for MUFA, the most dominant fatty acid was oleic acid (C18:1n9c), at 398.5 mg/100 g wet sample. Moonfish also contained significantly higher (Tukey post-hoc test, *P* < 0.05) of heptadecenoic acid (C17:1, 81.5 mg/100 g wet sample) compared to others (Table 3).

The high-fat fish, longtail shad also showed palmitic acid (C16:0) as the dominant fatty acid (12542.6 mg/100 g wet muscle, Table 2). For MUFA, longtail shad contained significantly higher (Tukey post-hoc test, *P* < 0.05) of palmitoleic acid (C16:1, 458.1 mg/100 g wet sample) and oleic acid (C18:1n9c, 2554.4 mg/100 g wet sample) compared to others (Table 3). While for PUFA, the fish contained significantly higher (Tukey post-hoc test, *P* < 0.05) of EPA (C20:5 n3, 2041.8 mg/100 g wet sample) and eicosatrienoicacid (C20:3n3, 437.5 mg/100 g wet sample) compared to others (Table 4). Besides, longtail shad was also high in *ω* − 6 fatty acids; with linolelaidic acid (C18:2n6t) at 2254.1 mg/100 g wet muscle, and linoleic acid (C18:2n6c) at 1327.4 mg/100 g wet muscle.

As in most of fish samples, shellfish including cuttlefish, cockles, and oyster also showed palmitic acid (C16:0) as the most prominent fatty acid (Table 2). Meanwhile, prawn showed caprylic acid (C8:0) as the highest fatty acid (139.9 mg/100 g wet sample), followed by palmitic acid (C16:0) at 125.3 mg/100 g wet sample. For MUFA, cockles contained higher oleic acid (C18:1n9c, 303.8 mg/100 g wet sample) compared to other shellfish (Table 3). While for PUFA, cockles were found to be higher in both EPA (C20:5n3, 343.0 mg/100 g wet sample) and DHA (C22:6n3, 123.4 mg/100 g wet sample), compared to other shellfish (Table 4). However, the content of alpha-linolenic acid (C18:3n3) in cockles (18.6 mg/100 g wet sample) was the lowest among all samples; with cuttlefish (251.1 mg/100 g wet sample), prawn (320.4 mg/100 g wet sample), and oysterr (100.7 mg/100 g wet sample).

### 3.2. Fatty Acids of Fish and Shellfish from Local and Other Countries

Most of previous local studies have focused on qualitative aspects of fat content in various samples of marine and freshwater origin samples [10–15]. A previous study found very high percentage of PUFA; with omega-3 PUFA (29.7–48.4%), other PUFA (27.7–40.0%), and omega-6 PUFA (11.0–20.0%) in ten common fish species with the current study [9]. Meanwhile, percentages of SFA and MUFA were quite low; at 3.63–11.4% and 1.37–9.12%, respectively. This study found hexadecadienoic acid as the most prominent fatty acid (18.1–24.9%) in six samples, hexadecatrienoic acid (C16:3n4) in two samples, and hexadecadienoic acid (C16:2) in other two samples [10]. Another local study reported PUFA as the dominant group of fatty acid in seven species, and SFA as the dominant fatty acid group in another six species of their fish samples [11]. Palmitic acid (C16:0) was the most prominent fatty acid (17.6–32.1%) in eight samples; meanwhile, DHA (C22:6n3) was the highest fatty acid (19.3–24.0%) in another five samples [11]. Same findings were reported by other study of 16 species of local pelagic fish; with palmitic acid (C16:0) as the most prominent fatty acid (20.9–34.5%) in nine species, meanwhile, DHA (C22:6n3) was the highest fatty acid (26.0–29.8%) in another seven species [12]. The current study, however, focuses on the quantitative aspect of samples fat thus allow limited comparison be made with these previous local data. However, there were a few studies focused on the quantitative aspects of fatty acids in other cold water species of marine and freshwater fish and shellfish performed in other countries. The comparisons made with the previous findings would be useful in giving better overview of the content of fatty acids in local marine fish and shellfish.

One of the previous quantitative studies of fatty acids had reported the *ω* − 3 and *ω* − 6 fatty acids content in different types (wild, farmed, supermarket, and feed) of salmon from North America and Europe [16]. Linoleic acid (C18:2n6) were at 67, 647, 604, and 1719 mg/100 g muscles for wild salmon, farmed salmon, supermarket salmon, and salmon feed [16]; which were higher than most of the samples (4.3–215.3 mg/100 g wet sample) in current study (Table 4). However, longtail shad is comparable in linoleic acid content with salmon feed, at 1327.4 mg/100 g wet sample [16]. This previous study also reported low *γ*-linolenic acid (C18:3n6) at 3 mg/100 g (wild salmon), 14 mg/100 g (farmed salmon), 13 mg/100 g (supermarket salmon), and 40 mg/100 g (salmon feed) [16]. Only three samples in current study were found to contain this fatty acid; hard tail scad (1.6 mg/100 g), silver pomfret (10.9 mg/100 g), and black pomfret (29.1 mg/100 g), which were comparable with their findings [16]. For *α*-linolenic acid (C18:3n3), most samples in current study contained about 100–300 mg/100 g of this fatty acid; which were higher than wild salmon (50 mg/100 g), comparable with farmed salmon (181 mg/100 g) and supermarket salmon (168 mg/100 g), meanwhile were lower when compared to salmon feed (505 mg/100 g) [16].

Most samples in current study contained lower EPA (C20:5n3, 2.7–343.0 mg/100 g wet sample) compared to wild salmon (414 mg/100 g), farmed salmon (1079 mg/100 g), supermarket salmon (969 mg/100 g), and salmon feed (2638 mg/100 g) [16]. Only longtail shad was found to contain comparable amount of EPA with salmon feed; at 2638 mg/100 g wet sample [16]. It is really interesting to find such a high level of EPA in longtail shad, as high intake of this fatty acid had been related with protective effects to the occurrence of asthma, coronary heart problems and many other diseases [17–19]. Meanwhile, compared to DHA values (629–2633 mg/100 g) reported by the previous study [16], the content of this fatty acid in most samples of the current study were lower (9.0–277.1 mg/100 g wet sample). However, yellowstripe scad (782.1 mg/100 g wet sample) was found to contain slightly higher DHA content compared to wild salmon (629 mg/100 g) [16].

Data from National Nutrient Data by United States Department of Agriculture (USDA) showed Greenland halibut, farmed catfish, wild catfish, farmed salmon, and wild salmon contained EPA + DHA at 1177.6, 177.6, 236.5, 2147.1, and 1840 mg/100 g muscles [20]. Generally, most samples in the current study contained EPA + DHA amounts (11.8–551.7 mg/100 g wet sample); which were lower compared to Greenland halibut, farmed salmon, and wild salmon; but comparable with farmed and wild catfish. However, longtail shad (2210 mg/100 g wet sample) was found to contain EPA + DHA at comparable amount with farmed and wild salmon [20]. This could be due to the high fat content of the fish (23.2% fat) [5].

The current study is novel as it provides new quantitative findings for warm water species of fish and shellfish. Moreover, the findings are of necessary representativeness, which resulted from systematic sampling procedures performed. Data from the current study is also highly important as it represents samples from the Straits of Malacca, which aligns the west coast of Peninsular Malaysia, the main contributor of marine landings production in the country which produced 50.16% of the total marine landings production of Malaysia and 67.34% of the marine landings production of peninsular areas (Department of Fisheries Malaysia, 2007).

### 3.3. Ratios of Polyunsaturated/Saturated (P/F) and *ω* − 3/*ω* − 6 Fatty Acids

Overall, seventeen species of samples contained SFA as the dominant group of fatty acid (Table 5), with palmitic acid (C16:0) as the highest fatty acid quantified in most of samples (Table 2). The high amount of palmitic acid is due to its function as a key metabolite in fish, and the level is not influenced by the diet [21]. Meanwhile, seven of the samples (golden snapper, Indian threadfin, malabar red snapper, long-tailed butterfly ray, large-scale tongue sole, yellowstripe scad, and prawn) contained PUFA as the dominant group of fatty acid. Generally, the PUFA to SFA (P/S) ratios of most of the samples of this study (Table 5) were above the value for Menhaden oil (0.58); as suggested by Food and Drug Association (FDA) as PUFA supplement [22]. Besides, ratios exceeding 0.50 have also been shown to lower blood cholesterol level [23].

Among the lean fish, hardtail scad and Indian mackerel contained fairly high SFA but fairly low PUFA; which resulted in low P/S ratio of 0.6 and 0.3, respectively. Meanwhile, other lean fish showed higher P/S ratios, between 0.6 and 1.4. For low-fat fish, four-finger threadfin showed low P/S ratio of 0.5. Other low-fat fish showed P/S ratio between 0.6 and 1.7. While for the medium fat fish, moonfish contained quite high P/S ratio of 1.0. The high-fat fish, longtail shad contained higher SFA content compared to PUFA that resulted in low P/S ratio of 0.4. Overall, most of samples contained favorable ratio of fatty acids, which was beneficial for PUFA intake and lowering blood cholesterol [22, 23].

There is no specific trend in levels of saturation or unsaturation relative to the fat content of samples. On the other hand, a very high positive correlation was found between fat content of samples with all three classes of fatty acids; SFA (Pearson's *r* = 0.989, *P* = 0.001), MUFA (Pearson's *r* = 0.984, *P* = 0.001), and PUFA (Pearson's *r* = 0.983, *P* = 0.001). This suggested that increment in fat content is followed by increment in the content of all forms of fatty acids: SFA, MUFA, and PUFA in the samples.

The −3 : *ω* − 6 ratio has been suggested as a useful indicator for comparing relative nutritional values of fish oils. The ratio is also expressed in the form of *ω* − 3/*ω* − 6. It has been suggested that an −3 : *ω* − 6 ratio of 1 : 1 to 1 : 5 (or *ω* − 3/*ω* − 6 between 0.2 and 1.0) would constitute a healthy human diet [22]. High *ω* − 3 : *ω* − 6 ratio is preferred, as excess *ω* − 6 fatty acids can counteract the health benefits of *ω* − 3 fatty acid intake [24].

Overall, long-tailed butterfly ray and cuttlefish had very high ratios of *ω* − 3/*ω* − 6, at 13.3 and 15.3, respectively (Table 5). However, the high ratio was not contributed to by high *ω* − 3 fatty acids, but by the low content of *ω* − 6 fatty acids. These fish contained 349.3 and 472.9 mg/100 g wet samples of *ω* − 3 fatty acids, respectively, which were within the common range of other samples. Meanwhile, their *ω* − 6 fatty acids contents were only 26.3 mg/100 g wet sample for long-tailed butterfly ray, and 30.9 mg/100 g wet sample for cuttlefish. The same reason applied to the fairly high ratio of *ω* − 3/*ω* − 6 at 9.8, 8.7, 7.8, and 7.3 for another four samples; namely, Indian threadfin, prawn, large-scale tongue sole, and Spanish mackerel. These samples contained *ω* − 3 fatty acids between 235.9 and 414.3 mg/100 g wet samples; meanwhile their *ω* − 6 fatty acids were below than 40 mg/100 g wet sample. While for yellowstripe scad, the high ratio of *ω* − 3/*ω* − 6 was contributed by its high content of *ω* − 3 fatty acids (1224.8 mg/100 g wet sample) compared to *ω* − 6 fatty acids (192.2 mg/100 g wet sample). In contrast, longtail shad contained high content of *ω* − 6 fatty, resulted in fairly low ratio of −3 : *ω* − 6 (0.8), which was the lowest among all samples.

### 3.4. Validation Procedures

A few validation analyses were performed to ensure the reliability of the method used for fatty acid analysis in this study; these measures included linearity, precision (repeatability and reproducibility), and recovery tests. Linear relationships were observed for myristic acid (C14:0) standard (linearity parameters: *y* = 221.25*x* − 23.04; *R* ^2^ = 0.999), heptadecanoic acid (C17:0) standard (linearity parameters: *y* = 7552*x* − 1060; *R* ^2^ = 0.9976), and linoleic acid (C18:2n6) standard (linearity parameters: *y* = 325.23*x* + 18.996; *R* ^2^ = 0.9985).

The precision test assessed both repeatability (within-day) and reproducibility (between-day) tests.  Table 6 shows the RSD values for the repeatability test of fatty acids; these values ranged between 1.4–3.6% for palmitic acid and 0.2–1.9% for linolenic acid. Meanwhile, Table 7 shows the RSD values for the reproducibility test, which ranged between 0.9–2.4% for palmitic acid, and 0.16–1.86% for linolenic acid. The RSD values in this study were lower compared to most RSD values (3.1–13.3%) as reported previously [25], but were higher when compared with excellent RSD values (<2.0%) reported by another study [26]. However, from overall, the RSD values in all repeatability and reproducibility tests were satisfactory and showed that the methods used were reliable to produce precise data for multiple determinations of fatty acids (saturated and unsaturated) in fish and shellfish samples, both in a single-day and multiple-day determinations.

Meanwhile, the percentages of recoveries for different concentrations of myristic acid were between 90.3–105.6% in golden snapper and 106.8–119.4% in cockles (Table 8). Meanwhile for heptadecanoic acid, the percentages of recoveries were between 87.1–111.9% in golden snapper and 112.8–115.9% in cockles. For stearic acid, the range of 93.4−108.8% in cockles. Overall, the recovery percentages were satisfactory, at about 90–120%, which is comparable to the values (most showed 80–120%) reported by previous studies [25, 27]. This shows that the methods used in the analysis are highly accurate for determinations of fatty acid contents at various concentrations in fish and shellfish samples.

## 4. Conclusion

Overall, most of the marine fish and shellfish samples contained desirable compositions of fatty acids with a fairly high amount of *ω* − 3 fatty acids, a suitable ratio of *ω* − 3/*ω* − 6 fatty acids, and preferable P/S ratios which were higher than the level in the recommended PUFA supplement, Menhaden oil. Three samples were identified as being outstanding in their desirable overall composition of fatty acids: yellowstripe scad (highest DHA, *ω* − 3/*ω* − 6 = 6.4, P/S = 1.7), moonfish (highest ALA, *ω* − 3/*ω* − 6 = 1.9, P/S = 1.0), and longtail shad (highest EPA, *ω* − 3/*ω* − 6 = 0.8, P/S = 0.4). These findings showed that marine fish and shellfish from warm water area contain a good composition of fatty acids and could provide many health benefits if consumed regularly. These reliable and representative data are very useful to develop a nutritional database of marine fish and shellfish from warm water area and as reference to people for intake locally and globally. Additionally, the findings also showed that some identified species of fish and shellfish from this area may have possible value in terms of future manipulation for various nutraceutical purposes. However, further studies should be developed on this matter.

## Figures and Tables

**Table 1:** List of samples with narrow range of weight, length, and fat content.

Category	Common name	Local name	Scientific name	Habitat	Range of weight (g)	Range of length (cm)	Fat content (%)
Lean fish (<2% fat)	Hardtail scad	Cencaru	*Megalapsis cordyla*	Pelagic	100–250	21–28	1.53 ± 0.15^a^
	Golden snapper	Jenahak	*Lutjanu sjohnii*	Demersal	490–510	30–35	1.29 ± 0.41^a^
	Indian mackerel	Kembung	*Rastrelliger kanagurta*	Pelagic	50–100	14–20	1.80 ± 0.62^a^
	Indian threadfin	Kurau	*Polynemus indicus*	Demersal	350–1450	36–59	0.85 ± 0.21^a^
	Malabar red snapper	Merah	*Lutjanus argentimeculatus*	Demersal	580–760	28–37	1.37 ± 1.10^a^
	Dorab wolfherring	Parang	*Chirocentrus dorab*	Pelagic	200–900	40–71	1.22 ± 0.22^a^
	Long-tailed butterfly ray	Pari	*Gymnura spp.*	Demersal	1300–1700	32–36	0.93 ± 0.12^a^
	Large-scale tongue sole	Sebelah/Lidah	*Cynoglossusarel*	Demersal	50–100	24–32	0.70 ± 0.10^a^
	Spanish mackerel	Tenggiri papan	*Scromberomorus guttatus*	Pelagic	200–450	30–42	1.05 ± 0.06^a^

Low fat fish (2–4% fat)	Black pomfret	Bawal hitam	*Parastromateus niger*	Pelagic	780–1040	33–42	2.33 ± 0.11^a^
	Silver pomfret	Bawal putih	*Pampus argentus*	Pelagic	100–200	15–25	2.09 ± 0.93^a^
	Sixbar grouper	Kerapu	*Epinephelus fasciatus*	Demersal	480–750	33–36	3.46 ± 3.46^ab^
	Japanese threadfin bream	Kerisi	*Nemipterus japonicus*	Demersal	100–230	18–25	2.70 ± 0.37^ab^
	Yellowstripe scad	Selar kuning	*Selaroides leptolepis*	Pelagic	50–100	16–20	2.12 ± 0.50^a^
	Gray eel-catfish	Sembilang	*Plotosus spp.*	Demersal	350–600	40–50	3.04 ± 0.59^ab^
	Fourfinger threadfin	Senangin	*Eleutheronema tetradactylum*	Pelagic	150–300	27–32	2.10 ± 0.25^a^
	Giant seaperch	Siakap	*Lates calcarifer*	Demersal	700–1000	38–42	2.68 ± 0.79^ab^
	Fringescale sardinella	Tamban	*Clupea fimbriata*	Pelagic	20–40	13–17	3.00 ± 2.40^ab^

Medium fat fish (4–8% fat)	Moonfish	Nyior-nyior	*Trachinotus blochii*	Demersal	400–1400	31–47	6.89 ± 2.76^b^

High fat fish (>8% fat)	Longtail shad	Terubuk	*Hilsa macrura*	Pelagic	928	40–45	23.15 ± 0.00^c^

Shellfish	Cuttlefish	Sotong	*Sepia officinalis*	—	20–45	12–18	1.35 ± 0.28^a^
	Prawn	Udang putih	*Metapenaeus affinis*	—	10–20	12–17	1.06 ± 0.10^a^
	Cockles	Kerang	*Anadara granosa*	—	10–20	2–5	1.93 ± 1.28^a^
	Oyster	Tiram	*Ostrea spp*.	—	100–300	14–48	1.24 ± 0.00^a^

^1^Categories of fish samples were based on the fat content [5].

^2^Different superscript lowercase letters (^a–c^) in the same column show significant differences at *P* < 0.05 (Tukey post-hoc test) [5].

**Table 2:** Saturated fatty acids (SFAs) content of samples.

Category	Samples	Saturated fatty acids (SFA) content (mg/100 g wet sample)								
		8:0	12:0	14:0	15:0	16:0	17:0	18:0	24:0	*∑*SFA
Lean fish (<2% fat)	Hardtail scad	42.2^ab^	1.4^abcd^	69.8^a^	18.6^abc^	531.7^a^	45.2^a^	nd	nd	708.8
	Golden snapper	152.9^bcd^	0.7^abc^	9.6^a^	8.5^ab^	203.7^a^	21.5^a^	1.8^a^	20.1^abcd^	418.7
	Indian mackarel	139.0^abcd^	0.7^abc^	64.1^a^	9.5^ab^	313.7^a^	22.8^a^	35.7^bcd^	2.3^ab^	587.8
	Indian threadfin	98.9^abcd^	0.9^abc^	10.3^a^	6.8^a^	134.7^a^	17.7^a^	4.8^ab^	33.4^bcde^	307.4
	Malabar red snapper	112.8^abcd^	0.9^abc^	28.8^a^	5.7^a^	373.7^a^	31.8^a^	nd	4.3^ab^	557.9
	Dorab wolfherring	72.2^abc^	1.4^abcd^	39.3^a^	7.6^a^	395.9^a^	21.1^a^	nd	nd	537.4
	Long-tailed butterfly ray	140.1^abcd^	nd	4.6^a^	2.9^a^	129.8^a^	11.3^a^	nd	nd	288.8
	Large-scale tongue sole	80.7^abc^	0.3^ab^	7.5^a^	5.4^a^	95.1^a^	15.2^a^	1.5^a^	36.8^cde^	242.4
	Spanish mackarel	86.3^abcd^	0.4^ab^	16.1^a^	5.9^a^	183.7^a^	9.5^a^	nd	21.0^abcde^	322.8

Low fat fish (2–4% fat)	Black pomfret	214.8^d^	nd	46.9^a^	18.0^abc^	549.5^a^	48.5^a^	6.9^ab^	50.4^def^	935.0
	Silver pomfret	40.0^ab^	1.1^abc^	102.4^a^	15.5^ab^	590.2^a^	38.6^a^	11.2^abc^	79.7^f^	878.7
	Sixbar grouper	186.1^cd^	1.6^abcd^	65.6^a^	19.8^abc^	775.6^a^	62.1^a^	6.4^ab^	191.6^g^	1308.7
	Japanese threadfin bream	34.0^ab^	5.2^de^	102.6^a^	28.2^abcd^	996.5^a^	36.8^a^	8.0^ab^	nd	1211.2
	Yellowstripe scad	101.1^abcd^	1.7^abcd^	69.5^a^	19.9^abc^	560.8^a^	65.7^a^	nd	10.9^ab^	829.6
	Gray eel-catfish	35.3^ab^	3.9^bcde^	159.7^a^	24.3^abc^	717.8^a^	359.0^a^	40.9^cd^	nd	1340.8
	Fourfinger threadfin	52.9^ab^	2.5^abcd^	127.2^a^	33.7^bcd^	677.0^a^	31.9^a^	7.0^ab^	21.1^abcde^	953.4
	Giant seaperch	79.5^abc^	4.4^cde^	102.5^a^	15.5^ab^	928.2^a^	76.7^a^	8.0^ab^	52.4^ef^	1267.2
	Fringescale sardinella	21.5^a^	2.2^abcd^	242.0^a^	43.1^cd^	822.2^a^	108.1^a^	33.5^bcd^	nd	1272.8

Medium fat fish (4–8% fat)	Moonfish	383.9^e^	7.2^e^	335.3^a^	53.4^d^	1308.0^a^	431.6^a^	31.1^abcd^	21.9^abcde^	2572.4

High fat fish (>8% fat)	Longtail shad	20.3^a^	21.1^f^	2805.4^b^	113.8^e^	12542.6^b^	2034.5^b^	82.5^e^	nd	17620.2

Shellfish	Cuttlefish	93.7^abcd^	0.7^abc^	30.9^a^	9.3^ab^	394.5^a^	7.0^a^	nd	33.0^bcde^	569.1
	Prawn	139.9^abcd^	0.3^ab^	7.0^a^	11.4^ab^	125.3^a^	24.5^a^	nd	nd	308.4
	Cockles	37.0^ab^	2.8^abcd^	69.9^a^	12.4^ab^	359.2*ª*	130.3*ª*	9.5^abc^	22.2^abcde^	643.3
	Oyster	30.2^ab^	0.5^abc^	41.4*ª*	10.0^ab^	325.8*ª*	80.3^a^	62.6^de^	13.0^abc^	563.7

^1^Categories of fish samples were based on the fat content [5].

^2^Different superscript lowercase letters (^a–g^) in same column show significant differences at *P* < 0.05 (Tukey post-hoc test).

^3^nd: not detected.

**Table 3:** Monounsaturated fatty acids (MUFAs) content of samples.

Category	Samples	Monounsaturated fatty acids (MUFAs) content (mg/100 g wet sample)					
		14:1	15:1	16:1	17:1	18:1 n9c	*∑*MUFA
Lean fish (<2% fat)	Hardtail scad	2.5^abc^	1.5^ab^	7.5^abc^	45.7^ef^	63.9^ab^	121.2
	Golden snapper	2.7^abc^	4.6^abc^	40.7^de^	6.6^ab^	39.5^a^	94.0
	Indian mackarel	1.8^abc^	7.5^c^	116.4^f^	10.4^ab^	158.7^ab^	294.8
	Indian threadfin	1.0^ab^	1.3^ab^	3.3^ab^	14.1^abc^	18.5^a^	38.3
	Malabar red snapper	1.0^ab^	1.0^ab^	4.4^ab^	16.0^abc^	119.5^ab^	141.8
	Dorab wolfherring	1.0^ab^	0.6^a^	1.1^a^	20.1^abcd^	123.1^ab^	145.9
	Long-tailed butterfly ray	1.6^abc^	0.8^a^	1.9^ab^	7.7^ab^	72.2^ab^	84.6
	Large-scale tongue sole	0.6^a^	1.4^ab^	nd	10.6^ab^	20.8^a^	33.4
	Spanish mackarel	0.4^a^	0.3^a^	2.8^ab^	13.8^abc^	53.2^ab^	70.6

Low fat fish (2–4% fat)	Black pomfret	1.8^abc^	4.1^abc^	13.6^abcd^	20.6^abcd^	98.8^ab^	138.8
	Silver pomfret	1.6^abc^	1.2^ab^	30.9^bcde^	36.0^cdef^	81.5^ab^	151.1
	Sixbar grouper	4.5^bcd^	15.7^d^	116.0^f^	nd	151.0^ab^	287.2
	Japanese threadfin bream	6.9^de^	1.5^ab^	105.3^f^	28.8^bcdef^	144.7^ab^	287.2
	Yellowstripe scad	2.8^abc^	1.9^ab^	43.4^e^	49.9^f^	188.8^ab^	286.8
	Gray eel-catfish	11.3^f^	5.7^bc^	nd	47.4^ef^	103.7^ab^	168.1
	Fourfinger threadfin	5.1^cd^	2.4^ab^	35.3^cde^	43.2^def^	241.5^ab^	327.4
	Giant seaperch	3.6^abcd^	2.6^ab^	43.4^e^	25.2^bcde^	155.3^ab^	230.0
	Fringescale sardinella	3.5^abcd^	3.5^abc^	53.0^e^	51.3^f^	192.0^ab^	303.3

Medium fat fish (4–8% fat)	Moonfish	9.5^ef^	14.8^d^	nd	81.5^g^	398.5^b^	504.2

High fat fish (>8% fat)	Longtail shad	10.7^ef^	nd	458.1^g^	36.1^cdef^	2554.4^c^	3059.3

Shellfish	Cuttlefish	0.8^ab^	2.62^ab^	4.0^ab^	17.2^abc^	89.5^ab^	114.2
	Prawn	0.7^a^	1.67^ab^	7.4^abc^	16.9^abc^	87.8^ab^	114.5
	Cockles	2.9^abc^	13.52^d^	nd	75.9^g^	303.8^ab^	396.1
	Oyster	0.7^ab^	2.65^ab^	nd	25.1^bcde^	53.9^ab^	82.4

^1^Categories of fish samples were based on the fat content [5].

^2^Different superscript lowercase letters (^a–g^) in same column show significant differences at *P* < 0.05 (Tukey post-hoc test).

^3^nd: not detected.

**Table 4:** Polyunsaturated fatty acids (PUFA) content of samples.

Category	Samples	Polyunsaturated fatty acids (PUFA) content (mg/100 g wet sample)							
		18:2 n6t	18:2 n6c	18:3 n6	18:3 n3	20:3 n3	20:5 n3	22:6 n3	*∑*PUFA
Lean fish (<2% fat)	Hardtail scad	22.5^a^	30.8^a^	1.6^a^	117.3^a^	nd	18.9^a^	196.0^cdefg^	387.0
	Golden snapper	105.2^ab^	9.6^a^	nd	365.6^ab^	nd	7.3^a^	18.6^ab^	506.3
	Indian mackarel	23.1^a^	29.3^a^	nd	53.9^a^	7.3^ab^	53.6^a^	23.3^ab^	190.5
	Indian threadfin	33.2^a^	5.2^a^	nd	271.7^a^	nd	23.7^a^	82.2^abcde^	415.9
	Malabar red snapper	86.1^ab^	60.4^a^	nd	335.0^ab^	9.2^ab^	24.1^a^	209.9^defg^	724.7
	Dorab wolfherring	36.3^a^	6.4^a^	nd	204.0^a^	11.6^ab^	23.9^a^	54.3^abcd^	336.5
	Long-tailed butterfly ray	11.2^a^	15.1^a^	nd	337.5^ab^	nd	2.7^a^	9.0^a^	375.5
	Large-scale tongue sole	25.9^a^	4.3^a^	nd	111.6^a^	2.5^ab^	8.3^a^	113.4^abcde^	266.2
	Spanish mackarel	18.2^a^	19.7^a^	nd	178.9^a^	nd	27.7^a^	69.8^abcde^	314.2

Low fat fish (2–4% fat)	Black pomfret	16.8^a^	45.1^a^	29.06^c^	272.8^a^	nd	73.5^a^	277.1^fg^	714.3
	Silver pomfret	104.7^ab^	49.5^a^	10.91^b^	136.2^a^	6.1^ab^	116.3^a^	148.0^abcdefg^	571.6
	Sixbar grouper	139.2^ab^	119.3^a^	nd	645.5^b^	nd	100.9^a^	197.7^cdefg^	1202.5
	Japanese threadfin bream	81.6^ab^	51.0^a^	nd	112.2^a^	nd	258.7^a^	293.0^g^	796.5
	Yellowstripe scad	78.6^ab^	113.6^a^	nd	338.1^ab^	7.5^ab^	97.1^a^	782.05^h^	1417.0
	Gray eel-catfish	257.4^ab^	133.6^a^	nd	185.0^a^	nd	145.8^a^	88.8^abcde^	810.7
	Fourfinger threadfin	148.3^ab^	48.7^a^	nd	114.3^a^	nd	96.2^a^	53.1^abc^	460.6
	Giant seaperch	142.5^ab^	94.4^a^	nd	245.6^a^	215.7^d^	139.5^a^	95.4^abcde^	933.0
	Fringescale sardinella	33.2^a^	164.2^a^	nd	69.3^a^	31.0^c^	211.5^a^	225.4^efg^	734.6

Medium fat fish (4–8% fat)	Moonfish	637.7^ab^	215.3^a^	nd	1046.8^c^	257.6^e^	176.7^a^	122.6^abcdef^	2456.7

High fat fish (>8% fat)	Longtail shad	2254.1^b^	1327.4^b^	nd	68.9^a^	437.5^f^	2041.8^b^	168.7^bcdefg^	6298.4

Shellfish	Cuttlefish	13.4^a^	17.5^a^	nd	251.1^a^	14.9^b^	121.9^a^	85.0^abcde^	503.8
	Prawn	16.3^a^	31.3^a^	nd	320.4^ab^	nd	48.7^a^	45.3^abc^	461.9
	Cockles	71.5^ab^	55.0^a^	nd	18.6^a^	nd	343.0^a^	123.4^abcdef^	611.4
	Oyster	62.0^a^	35.9^a^	nd	100.7^a^	nd	54.0^a^	82.8^abcde^	335.4

^1^Categories of fish samples were based on the fat content [5].

^2^Different superscript lowercase letters (^a–h^) in same column show significant differences at *P* < 0.05 (Tukey post-hoc test).

^3^nd: not detected.

**Table 5:** Polyunsaturated/saturated fatty acids (P/S) and *ω* − 3/*ω* − 6 ratio of samples.

Classification	Samples	SFA	MUFA	PUFA	PUFA/SFA	*ω* − 3	*ω* − 6	*ω* − 3/*ω* − 6
Lean fish (<2% fat)	Hardtail scad	708.8	121.2	387.0	0.5	332.1	54.8	6.1
	Golden snapper	418.7	94.0	506.3	1.2	391.5	114.8	3.4
	Indian mackarel	587.8	294.8	190.5	0.3	138.1	52.4	2.6
	Indian threadfin	307.4	38.3	415.9	1.4	377.5	38.4	9.8
	Malabar red snapper	557.9	141.8	724.7	1.3	578.3	146.5	3.9
	Dorab wolfherring	537.4	145.9	336.5	0.6	293.7	42.7	6.9
	Long-tailed butterfly ray	288.8	84.6	375.5	1.3	349.3	26.3	13.3
	Large-scale tongue sole	242.4	33.4	266.2	1.1	235.9	30.2	7.8
	Spanish mackarel	322.8	70.6	314.2	1.0	276.3	37.9	7.3

Low fat fish (2–4% fat)	Black pomfret	935.0	138.8	714.3	0.8	623.3	91.0	6.8
	Silver pomfret	878.7	151.1	571.6	0.7	406.6	165.1	2.5
	Sixbar grouper	1308.7	287.2	1202.5	0.9	944.1	258.5	3.7
	Japanese threadfin bream	1211.2	287.2	796.5	0.7	663.9	132.6	5.0
	Yellowstripe scad	829.6	286.8	1417.0	1.7	1224.8	192.2	6.4
	Gray eel-catfish	1340.8	168.1	810.7	0.6	419.6	391.1	1.1
	Fourfinger threadfin	953.4	327.4	460.6	0.5	263.6	197.0	1.3
	Giant seaperch	1267.2	230.0	933.0	0.7	696.2	236.9	2.9
	Fringescale sardinella	1272.8	303.3	734.6	0.6	537.2	197.4	2.7

Medium fat fish (4–8% fat)	Moonfish	2572.4	504.2	2456.7	1.0	1603.7	853.0	1.9

High fat fish (>8% fat)	Longtail shad	17620.2	3059.3	6298.4	0.4	2716.9	3581.5	0.8

Shellfish	Cuttlefish	569.1	114.2	503.8	0.9	472.9	30.9	15.3
	Prawn	308.4	114.5	461.9	1.5	414.3	47.6	8.7
	Cockles	643.3	396.1	611.4	1.0	484.9	126.5	3.8
	Oyster	563.7	82.4	335.4	0.6	237.5	97.9	2.4

^1^Categories of fish samples were based on the fat content [5].

**Table 6:** Repeatability (within-day precision) data of fatty acids (*n* = 4).

Sample	*n*	Palmitic acid (C16:0)					Linolenic acid (C18:3 n3)				
		Mean of fatty acid content (mg/g oil)	SD	RSD (%)	Minimum	Maximum	Mean of fatty acid content (mg/g oil)	SD	RSD (%)	Minimum	Maximum
Gray eel-catfish	4	321.06	4.60	1.43	315.91	326.43	55.40	0.19	0.34	55.23	55.66
Giant seaperch	4	334.48	12.04	3.59	319.45	345.48	92.50	1.73	1.87	91.00	94.02
Cockles	4	146.36	3.00	2.05	142.12	149.19	109.22	1.86	1.70	107.52	111.33

**Table 7:** Reproducibility (between-day precision) data of fatty acids (*n* = 3).

Sample	*n*	Palmitic acid (C16:0)					Linolenic acid (C18:3 n3)				
		Mean of fatty acid content (mg/g oil)	SD	RSD (%)	Minimum	Maximum	Mean of fatty acid content (mg/g oil)	SD	RSD (%)	Minimum	Maximum
Gray eel-catfish	3	319.27	3.48	1.09	315.91	322.85	55.31	0.09	0.16	55.23	55.40
Giant seaperch	3	339.49	8.19	2.41	330.15	345.48	91.99	1.71	1.86	91.00	93.96
Cockles	3	147.77	1.25	0.85	146.84	149.19	108.51	1.48	1.36	107.52	110.22

**Table 8:** Recovery rates of fatty acids in samples.

Fatty acids standards	Concentrations (mg/mL)	Samples	Recovery (%)
Myristic (C14:0)	0.4	a	105.61
		b	106.84
	2.0	a	105.40
		b	119.35
	4.0	a	90.34
		b	114.80

Heptadecanoic (C17:0)	0.4	a	94.63
		b	114.13
	2.0	a	111.92
		b	112.84
	4.0	a	87.09
		b	115.92

Stearic (C18:0)	0.4	a	116.15
		b	106.75
	2.0	a	105.40
		b	108.81
	4.0	a	110.39
		b	93.39
